# The roles of musical expertise and sensory feedback in beat keeping and joint action

**DOI:** 10.1007/s00426-019-01156-8

**Published:** 2019-02-25

**Authors:** Benjamin G. Schultz, Caroline Palmer

**Affiliations:** 1 Department of Psychology, McGill University, Montreal, QC Canada; 2 Department of Psychopharmacology and Neuropsychology, Faculty of Psychology and Neuroscience, Maastricht University, Universiteitssingel 40, 6229 ER Maastricht, Netherlands

## Abstract

Auditory feedback of actions provides additional information about the timing of one’s own actions and those of others. However, little is known about how musicians and nonmusicians integrate auditory feedback from multiple sources to regulate their own timing or to (intentionally or unintentionally) coordinate with a partner. We examined how musical expertise modulates the role of auditory feedback in a two-person synchronization–continuation tapping task. Pairs of individuals were instructed to tap at a rate indicated by an initial metronome cue in all four auditory feedback conditions: no feedback, self-feedback (cannot hear their partner), other feedback (cannot hear themselves), or full feedback (both self and other). Participants within a pair were either both musically trained (musicians), both untrained (nonmusicians), or one musically trained and one untrained (mixed). Results demonstrated that all three pair types spontaneously synchronized with their partner when receiving other or full feedback. Moreover, all pair types were better at maintaining the metronome rate with self-feedback than with no feedback. Musician pairs better maintained the metronome rate when receiving other feedback than when receiving no feedback; in contrast, nonmusician pairs were worse when receiving other or full feedback compared to no feedback. Both members of mixed pairs maintained the metronome rate better in the other and full feedback conditions than in the no feedback condition, similar to musician pairs. Overall, nonmusicians benefited from musicians’ expertise without negatively influencing musicians’ ability to maintain the tapping rate. One implication is that nonmusicians may improve their beat-keeping abilities by performing tasks with musically skilled individuals.

## The role of musical expertise and sensory feedback in beat keeping and joint action

Imagine a music performance in which several musicians produce a synchronous and esthetically pleasing piece of music. Now, imagine that the musicians are unable to hear each other and each instrument slowly drifts out of time from the rest of the group. The second example is likely to be far less esthetically pleasing. This illustrates the importance of being able to keep time within a group setting and demonstrates that auditory feedback is, arguably, crucial to enable temporal coordination and synchrony between performers. We define auditory feedback as the sound outcomes of one’s own actions and also the sound productions from an external source, for example, a co-performer. Despite a growing body of literature on joint action (see Palmer, 2013; Repp & Su, 2013; Sebanz & Knoblich, 2009 for reviews) and sensorimotor integration (Repp, 2005), little is known about how people use and integrate auditory feedback from multiple sources to regulate their own timing and achieve interpersonal coordination. Moreover, the role of musical expertise in dyadic tapping is rarely explored and, therefore, the influence of the amount of training previously acquired by each individual within a pair remains unknown. The present study investigated how musicians and nonmusicians use auditory feedback of their own sound productions and the sound productions of their partner to coordinate in time and maintain a regular rate.

One way in which people temporally coordinate actions is through entrainment to temporal regularities such as the beat (London, 2012). The beat is a regular pulse that can be perceptually abstracted from auditory signals (e.g., music or speech) or dynamic visual signals (e.g., hand/finger movements or moving objects; Hove & Keller, 2010; Hove, Spivey, & Krumhansl, 2010; Luck & Nte, 2007). Tempo refers to the beat rate whereby a faster tempo is an increase in the rate of beats. The dynamic attending theory (Jones & Boltz, 1989) proposes that the beat is abstracted through entrainment, whereby neural oscillations synchronize with and adapt to an external signal. This adaptive process allows a perceiver to abstract the beat from, and synchronize with, various signals including isochronous (evenly-spaced) metronomes, tempo-varying metronomes (Large, Fink, & Kelso, 2002), simple and complex rhythms (Nozaradan, Peretz, & Mouraux, 2012), dynamic speech (Schultz et al., 2016) and rich musical sequences (Large, 2000). Moreover, there is evidence that humans spontaneously move to the beat of music and in synchrony with others, suggesting that synchronizing with music and with co-performers may play a role in social cohesion (Bolt, Poncelet, Schultz, & Loehr, 2016; Demos, Chaffin, Begosh, Daniels, & Marsh, 2012; Hove & Risen, 2009; Janata, Tomic, & Haberman, 2012; Stupacher, Maes, Witte, & Wood, 2017). This phenomenon is called spontaneous synchronization and can occur either intentionally (e.g., Bolt et al., 2016) or unintentionally (e.g., Demos et al., 2012). We investigated how humans use auditory feedback from their own sound productions and the sound productions of a partner to maintain a regular beat during dyadic tapping. We also examined whether partners unintentionally synchronize with each other when they hear their partner’s sound productions, but are instructed to maintain a regular beat regardless of the timing of their partner’s sound productions.

Success in joint action tasks is hypothesized to require the ability to monitor the timing of one’s own actions to predict or track the timing of another person’s action (Keller, Novembre, & Hove, 2014; Sebanz & Knoblich, 2009). Some have proposed that individual differences in interpersonal synchronization abilities are related to individuals’ spontaneous rates of movement (Loehr & Palmer, 2011; Zamm, Wellman, & Palmer, 2016), their ability to predict the timing of stimulus onsets (Mills, van der Steen, Schultz, & Keller, 2015; Pecenka & Keller, 2011), equality of social status (Demos, Carter, Wanderley, & Palmer, 2017), musical imagery (Keller & Appel, 2010), or their musical expertise (Franěk, Mates, Radil, Beck, & Pöppel, 1991; Krause, Pollok, & Schnitzler, 2010). Such individual differences are thought to help regulate one’s own timing as well as predicting and tracking the timing of others’ actions. Individual differences may also exist in how people rely on sensory feedback in joint action tasks, namely, how they regulate their own movements based on their own sensory feedback, how they synchronize with sensory feedback produced by a partner, and how they integrate and compare the two types of sensory feedback (Shaffer, 1982). We compared musicians and nonmusicians to examine how musical expertise affects the way in which people use auditory feedback from themselves or from a partner to maintain a regular beat.

The role of one’s own sound productions has been examined extensively through manipulations of temporal delays or pitch shifts in auditory feedback (e.g., Aschersleben & Prinz, 1997; Pfordresher, 2003). The influence of auditory feedback in maintaining a tempo has received less attention. Repp (2006) examined how well expert musicians are able to maintain a tempo by themselves with and without auditory feedback from their own taps in the presence and absence of an auditory distractor sequence. Auditory feedback did not consistently improve the ability to keep the tempo, but it decreased overall variability in the tap onsets for a slower tempo condition. Moreover, the presence of a distractor sequence increased the variability of the produced tempo. These results suggest that auditory feedback of one’s own sound productions may not be important for maintaining the tempo. However, the software and equipment used to produce auditory feedback may have introduced temporal delays and variability that led performers to ignore their sound productions (Schultz, 2018; Schultz & van Vugt, 2015). To overcome this problem, the present study used a device to record taps and present low-latency auditory feedback with low variability. We expect that both musicians and nonmusicians are better able to maintain a tempo with self-generated auditory feedback than with no auditory feedback, and that musicians are more accurate than nonmusicians at maintaining the metronomic beat.

The current study utilizes a tapping task to permit comparison between musicians and nonmusicians. To date, few experiments have examined the role of auditory feedback in dyadic tapping (Konvalinka, Vuust, Roepstorff, & Frith, 2010; Mates, Radil, & Pöppel, 1992; Nowicki, Prinz, Grosjean, Repp, & Keller, 2013). Mates and colleagues (1992) presented isochronous metronomes to tapping dyads (participants not chosen for musicianship) under four feedback conditions: no feedback, their own feedback (i.e., self-feedback), their partners’ feedback in the absence of their own feedback (i.e., other feedback), and a condition in which one participant heard their own feedback and their partner’s feedback while the other participant received no feedback. Results did not demonstrate mutual adaptation between co-performers in conditions where they could hear their partner. However, the metronome was present throughout each trial and, therefore, it is possible that participants coordinated with the metronome and ignored the more variable sequence of their partners’ feedback. Nowicki and colleagues (2013) asked tapping dyads (musicians) to alternate taps with their partner to an isochronous metronome under conditions of no feedback, self-feedback, other feedback, and full feedback. Results showed that asynchronies with the metronome were smaller with self- and full feedback than with no feedback, were larger with other feedback alone than with self-feedback and full feedback, and were larger for full feedback than self-feedback. Nowicki and colleagues suggested that asynchronies produced by an individual were only weakly influenced by the auditory feedback from a partner. One explanation for weak mutual influences is that participants were able to rely on the presence of the metronome and the partner’s feedback simply provided a type of temporal perturbation (see Repp, 2006). The present study employed a synchronization–continuation task in which the metronome stopped after the synchronization phase to investigate how co-performers mutually adapt in the absence of a reliable metronomic signal, and to examine how well tapping dyads can maintain the cued tempo.

Konvalinka et al. (2010) used such a synchronization–continuation paradigm to examine the influences of auditory feedback on tapping dyads’ mutual adaptation. Feedback conditions were: self-feedback, full feedback, and two conditions where the pair only heard the feedback of one person, that is, one participant received self-feedback and the second participant received other feedback. Results showed that participants were worse at maintaining the metronome tempo when provided with other feedback or full feedback than with self-feedback. However, the musical experience of participants was not reported and, assuming that participants were nonmusicians, it is therefore possible that participants lacked the necessary skills to maintain a regular beat in coordination with a partner. Based on the superior time-keeping abilities of expert musicians (Drake & Palmer, 2000), we expect that musicians maintain a tempo better than nonmusicians even when presented with feedback from a partner (i.e., other or full feedback). Konvalinka and colleagues (2010) also found that participants adapted to their partner when they received other feedback or full feedback. However, the task instruction was to both maintain the given tempo and to synchronize with their partner. To investigate unintentional synchronization within dyadic tapping, the present study used a synchronization–continuation task and only asked participants to maintain the metronome rate.

In a joint music performance context, Goebl and Palmer (2009) examined how duet pianists use auditory feedback to perform synchronously. Pianists performed with self-feedback, full feedback, or a feedback condition in which one participant heard self-feedback and the other participant heard self- and other feedback. Pianists produced smaller asynchronies and demonstrated greater adaptation in conditions in which performers could hear their partner, a result that is corroborated by those of Konvalinka and colleagues (2010) with (presumably) nonmusicians in a tapping task. Therefore, we expect that both musicians and nonmusicians unintentionally coordinate their actions when they can hear feedback from their partner. The present study examined how musicians and nonmusicians maintain a metronomic beat rate and unintentionally synchronize with their partner under two conditions of self-feedback (off, on) and two conditions of other feedback (off, on) in the auditory domain. Effectively, this produced four auditory feedback conditions: no feedback, self-feedback (only), other feedback (only), and full feedback (both self and other) (see Table 1).

**Table 1:** Design of feedback conditions in experiments

Self	Other	
	Off	On
Off	No feedback	Other feedback only
On	Self-feedback only	Full feedback

## Hypotheses

Two experiments addressed the roles of musical expertise and auditory feedback in temporal coordination during a dyadic synchronization–continuation tapping task. Participants, who could not see each other, were instructed to continue tapping at the metronomic rate when the metronome sound stopped (regardless of feedback from their partner’s taps). Experiment 1 assessed how musicians and nonmusicians maintain a regular beat under different feedback conditions when paired with a fellow musician or nonmusicians, respectively. Experiment 2 assessed how musicians and nonmusicians maintain a regular beat under different feedback conditions when pairs consisted of one musician and one nonmusician. Both experiments employed a 2 (self-feedback; off, on; within-subjects) by 2 (other feedback; off, on; within-subjects) x 3 (tempo; fast = 160 beats per minute [bpm], moderate = 120 bpm, slow = 96 bpm; within-subjects) design. Musical training (musician, nonmusician) was included as a between-subjects factor. Temporal coordination with the metronome and also with their partner during the continuation phase of each trial was measured using time series analysis methods (i.e., cross-correlations). Based on the results of Konvalinka et al. (2010), we expect that nonmusician pairs are less accurate at maintaining a tempo under conditions of other and full feedback, compared to self-feedback. We hypothesize that musicians and nonmusicians are more accurate at maintaining the tempo when in the presence of self-, other, and full feedback compared to no feedback. We also hypothesize that musicians are better than nonmusicians at maintaining the tempo when presented with other and full feedback. Finally, we hypothesize that both musicians and nonmusicians unintentionally synchronize with their partner in the other and full feedback conditions, and that musician pairs synchronize more than nonmusician pairs.

## Experiment 1

### Method

#### Participants

Musicians (*N* = 40) had six or more years of formal training on an instrument (*M* = 10.45 years, SD = 3.83, range = 6 to 23 years) and consisted of 22 females (18 males) with a mean age of 20.63 years (SD = 3.67, range 17 to 36 years). Nonmusicians (*N* = 40) had less than 3 years of formal training on an instrument (*M* = 0.71 years, SD = 0.91, range = 0 to 2 years) and consisted of 23 females (17 males) with a mean age of 23.05 years (SD = 7.16, range 18 to 48 years). All participants were recruited from McGill University. No participant reported any hearing impairment.

#### Apparatus

**Fig. 1:**
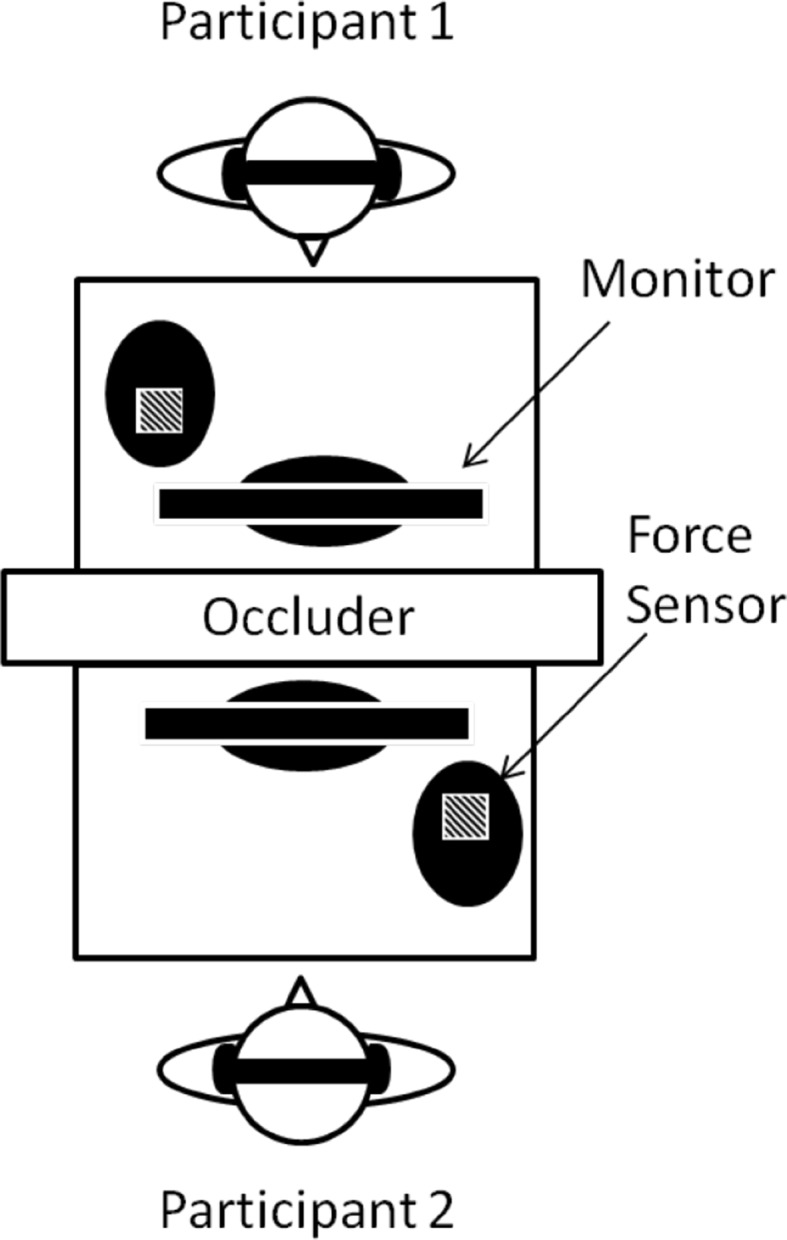
Experimental setup for the joint tapping task

Participants were each seated in front of a Dell Monitor with a visual occluder between them (see Fig. 1). Taps were recorded using a force sensitive resistor pad (1.72″ × 1.72″ Interlink FSR) connected to an Arduino and a custom Python script received the data via the serial port (see Schultz & van Vugt, 2015). The Arduino was connected to a wave shield that produced a sound with a mean latency of 2.6 ms (SD = 0.3) when the sensor recorded a tap. Auditory feedback was delivered to participants through Bose QC20 headphones. To control auditory feedback, the sounds produced by taps were routed through a MOTU 828 MkII external sound driver and distributed to participants through Max/MSP (Puckette & Zicarelli, 1990) according to the feedback condition. Sounds produced by taps and the auditory stimuli (i.e., metronomes) were recorded in Cubase, regardless of whether participants received auditory feedback. To prevent participants from hearing the sounds resulting from finger contact with the force sensor resistor, participants wore Bose QC20 noise-canceling earphones. Each participant tapped on a different table to prevent the use of vibro-tactile information emanating from their partner’s taps. Custom Matlab (Matlab, 1999) scripts were used to present instructions and stimuli to participants.

#### Procedure

Participants responded to advertisements and volunteered their degree of musical training prior to scheduling the experiment session to ensure pairs were correctly assigned to the musician and nonmusician groups. Upon arrival, informed consent was first obtained for both participants (REB: 404–0313). After completing a demographic questionnaire, participants performed the dyadic synchronization–continuation task. Participants were instructed to synchronize with an isochronous metronome for 12 events and, when the metronome stopped after the 12th event, to continue tapping at the same rate as the metronome until signaled to stop as indicated by a bongo drum sound. Participants were not instructed to synchronize with their partner but, instead, were told to “Synchronize with the metronome for 12 beats then continue to tap at the same rate as the metronome when it stops.” The trial continued for 20 uncued beats after the metronome stopped until the bongo drum sound signaled the end of the trial. There were three metronome tempi of 160 bpm (fast), 120 bpm (medium), and 96 bpm (slow) and tempi were performed twice within each feedback condition in a pseudorandom order. To prevent entrainment-based improvement between trials, no tempo was repeated twice consecutively [cf. (Large & Jones, 1999)]. There were four blocks of feedback and participants were informed of which feedback condition they were performing at the beginning of each block. The four feedback conditions were: no feedback, feedback from self, feedback from other, and feedback from self and other. The order of feedback conditions was counterbalanced across pairs. To aid participants in differentiating their feedback from that of their partner, one participant received a high tone (20 ms sinewave, 880 Hz, 5 ms ramp/damp) and the other received a low tone (20 ms sinewave, 220 Hz, 5 ms ramp/damp), and the tone-to-participant correspondence was counterbalanced within participants in two blocks.

At the start of the experiment and after the tone-to-participant correspondence was changed halfway through the experiment, participants were given an opportunity to familiarize themselves with the auditory feedback by freely tapping in each of the four feedback conditions with the metronome absent. Participants were informed that they would both receive the same metronome and, prior to each block, were told which feedback condition they would be performing. Participants then received practice trials of the feedback conditions until they demonstrated that they understood the task. Then participants performed all feedback conditions twice in a blocked design. After completing the first half of the experiment (four feedback conditions), participants completed a questionnaire on their musical experience. Then participants repeated the experimental conditions, but swapped who received the high and low tones corresponding to their taps. Altogether, the experiment consisted of four feedback conditions, three tempi, two tone-to-participant mappings, and two repetitions of each combination resulting in 48 trials. Each synchronization–continuation trial had approximately 16 s duration (depending on the tempo condition) and experiment sessions did not exceed 70 min.

#### Statistical analysis

To analyze temporal coordination, the discrete tapping onset times as well as the implied metronome beat onsets in each trial were transformed into continuous time series using the discrete to dynamic oscillator conversion (DiscDOC) toolbox in MATLAB [see Demos et al., 2012; Schultz & Demos, In preparation.]. The transformation interpolates the intervals between successive pair of tapped onsets with a sinusoid that has a period identical to that of the inter-onset interval between the two tap onsets. The transformation also interpolates the intervals between each pair of implied metronome onsets with a sinusoid with a fixed period identical to that of the tempo for that trial. The dependent variable was the cross-correlation coefficient between the transformations for (1) the implied metronome onsets, that is, the metronome beat should it have continued after the synchronization phase, and (2) the tap onsets of each participant within a pair. As cross-correlation coefficients were negatively skewed, an exponential transformation was applied to the data prior to analysis.

For comparisons with the hypothesized metronome, the time series for each participant within a pair were cross-correlated with the time series of the hypothesized metronome. The two cross-correlation coefficients were included in a linear mixed-effects model (LMEM) with pair coordination (i.e., cross-correlation coefficients between taps from the two participants) included as a predictor to account for partner influences on individual coordination with the implied metronome at each level of the within-subjects factors. For the coordination between partners, cross-correlation coefficients were entered twice per pair and each individual’s coordination with the metronome was entered as a predictor to account for the effect of individual performance in each pair at each level of the within-subjects factors. The LMEM for each dependent variable contained within-subjects fixed effects Self-feedback (2; off, on), Other feedback (2; off, on), the between-subjects effect musical training (2; musician, nonmusician), and the random effects participant nested within pair, and tempo (3; fast, medium, slow).

Data were preprocessed using MATLAB and analyzed using R statistical software (R Core Team, 2013). LMEMs were performed using the *nlme* library (Pinheiro, Bates, DebRoy, Sarkar, & R Core Team, 2015) and *F* statistics, significance values, and effect sizes (generalized eta squared; $\eta _{{\text{g}}}^{2}$ where 0.02 is small, 0.13 is medium, and 0.26 is large; Bakeman, 2005) were calculated using Satterthwaite approximation for degrees of freedom. Pairwise comparisons were obtained using Tukey’s honestly significant difference (HSD) from the *multcomp* library (Horthorn et al., 2017).

### Results

#### Synchronization with the cued tempo

For the cross-correlations between individuals’ taps and the implied metronome, there were significant main effects of Self-feedback [*F* (1, 872.63) = 7.40, *p* = 0.007, $\eta _{{\text{g}}}^{2}$ = 0.01], Other feedback [*F* (1, 302.81) = 15.27, *p* < 0.001, $\eta _{{\text{g}}}^{2}$ = 0.006], and Musical expertise [*F* (1, 46.14) = 25.35, *p* < 0.001, $\eta _{{\text{g}}}^{2}$ = 0.21]. There were also significant interactions between Self-feedback and Other feedback [*F* (1, 876.21) = 37.79, *p* < 0.001, $\eta _{{\text{g}}}^{2}$ = 0.06], between Other feedback and Musical expertise [*F* (1, 300.97) = 23.12, *p* < 0.001, $\eta _{{\text{g}}}^{2}$ = 0.08], and between Self-feedback, Other feedback, and Musical expertise [*F* (1, 876.38) = 17.82, *p* < 0.001, $\eta _{{\text{g}}}^{2}$ = 0.03]. No other interactions reached significance (*ps* > 0.38). Hence, we proceeded to investigate musicians and nonmusicians separately, and then compared musicians and nonmusicians in the different feedback conditions.

##### Musicians

**Fig. 2:**
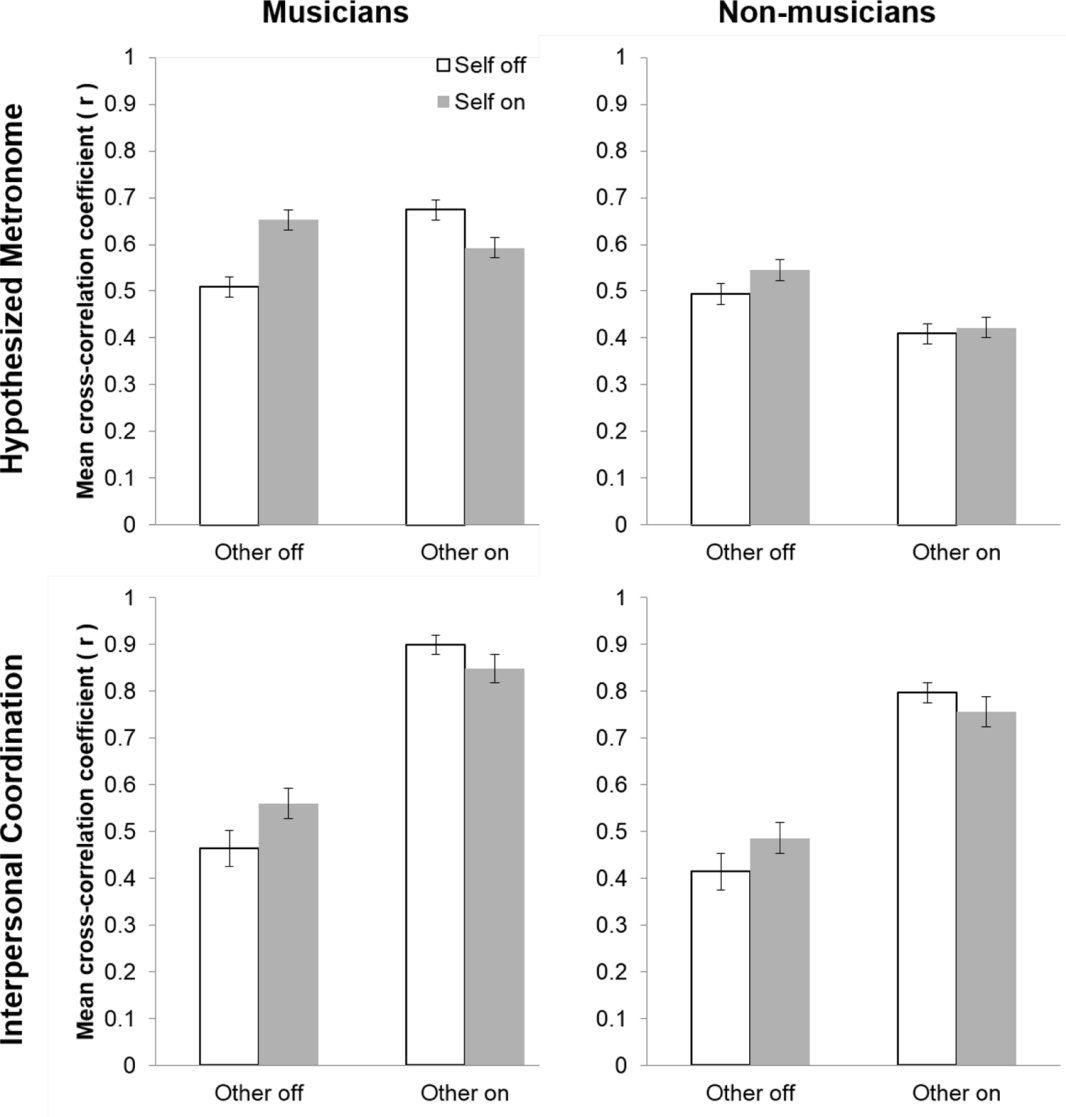
Mean cross-correlation coefficients between the wave transformations of tap onsets and the hypothesized metronome (top panels) and between the wave transformations of tap onsets of the pairs (bottom panels) for feedback conditions in Experiment 1 for musicians (left panels) and nonmusicians (right panels)

Planned comparisons were conducted to examine the interaction between Self-feedback and Other feedback for musicians. As shown in Fig. 2, top-left panel, the no feedback condition (Self off, Other off) produced significantly smaller cross-correlation coefficients than those in the self-feedback (Self on, Other off; *p* < 0.001) and other feedback (Self off, Other on; *p* < 0.001) conditions, but not the full feedback condition (Self on, Other on; *p* = 0.38)^1^. The self-feedback condition (Self on, Other off) was not significantly different from the other feedback condition (Self off, Other on; *p* = 1.00) and was significantly greater than the full feedback condition (Self on, Other on; *p* = 0.001). The other feedback condition (Self off, Other on) was significantly greater than the full feedback condition (Self on, Other on; *p* < 0.001). These results support the hypothesis that musicians use auditory feedback from both oneself and others to maintain the cued tempo. However, when both sources of auditory feedback were available, musicians did not perform significantly better than the condition with no auditory feedback.

##### Nonmusicians

Planned comparisons were conducted to explore the interaction between Self- and Other feedback for nonmusicians. As shown in Fig. 2, top right panel, cross-correlation coefficients for the no feedback condition (Self off, Other off) were significantly smaller than those for the self-feedback condition (Self on, Other off; *p* = 0.02) but were significantly larger than those for the other feedback (Self off, Other on; *p* < 0.001) and full feedback (Self on, Other on; *p* < 0.001) conditions. Similarly, the self-feedback condition demonstrated significantly larger cross-correlation coefficients than the other feedback (Self off, Other on; *p* < 0.001) and full feedback (Self on, Other on; *p* < 0.001) conditions. Cross-correlation coefficients did not significantly differ between the other feedback (Self off, Other on) and full feedback (Self on, Other off) conditions (*p* = 0.83). These results support the hypothesis that nonmusicians use self-feedback to maintain the cued tempo, but do not support the hypothesis that nonmusicians use auditory feedback from a (nonmusician) partner to maintain a beat; nonmusicians were significantly worse at maintaining the tempo when presented with the sound productions of their partner.

##### Musicians and nonmusicians comparison

To further investigate differences between musicians and nonmusicians in feedback conditions, planned comparisons were conducted between musicians and nonmusicians within the four feedback conditions. As shown in Fig. 2 (top panels), there was no significant difference between musicians and nonmusicians for the no feedback condition (Self off, Other off; *p* = 0.37), but musicians were significantly better at maintaining the cued tempo than nonmusicians for all other feedback conditions (*ps* < 0.003). These results partially support the hypothesis that musicians are better at maintaining the metronomic beat than nonmusicians; musicians were only better than nonmusicians when auditory feedback of self and/or other was present.

#### Interpersonal coordination

For the cross-correlations for partners within a pair, there were significant main effects of Other feedback [*F* (1, 900.93) = 1230.88, *p* < 0.001, $\eta _{{\text{g}}}^{2}$ = 0.62] and Musical expertise [*F* (1, 37.63) = 8.82, *p* = 0.005, $\eta _{{\text{g}}}^{2}$ = 0.09], and a significant interactions between Self-feedback and Other feedback [*F* (1, 909.25) = 35.63, *p* < 0.001, $\eta _{{\text{g}}}^{2}$ = 0.05] and between Other feedback and Musical expertise [*F* (1, 901.38) = 8.60, *p* = 0.003, $\eta _{{\text{g}}}^{2}$ = 0.01]. The main effect of Self-feedback and other interactions did not reach significance (*ps* > 0.44). The main effect of Musical expertise indicated that musician pairs produced larger cross-correlation coefficients than nonmusician pairs (see Fig. 2, bottom panels). Planned comparisons between musician pairs and nonmusician pairs within the four feedback conditions indicated that musician pairs only produced significantly greater cross-correlation coefficients than nonmusician pairs in the other feedback only condition (Self off, Other on; *p* < 0.001) and the full feedback condition (Self on, Other on; *p* = 0.02) but not in the two Other off conditions (Self on and Self off; *ps* > 0.11).

Planned comparisons were performed between feedback conditions to test the hypothesis that musicians and nonmusicians unintentionally synchronize with their partner when they can hear other feedback. As shown in Fig. 2, cross-correlation coefficients were larger in the Other feedback on condition compared to the Other feedback off condition for musicians and nonmusicians (ps < 0.001). This result supports the hypothesis that musicians and nonmusicians unintentionally synchronize with their partner when they hear the sound productions of that partner (even when the task and instruction does not require synchronization with the partner). Moreover, cross-correlation coefficients were significantly higher for musicians compared to nonmusicians in the Other feedback on condition (*p* = 0.002) but did not significantly differ in the Other feedback off condition (*p* = 0.20), suggesting that musicians synchronize with each other more than nonmusicians.

### Discussion

Experiment 1 revealed that the ability to maintain a beat in the presence of auditory feedback from oneself and a partner is influenced by musical expertise. Musicians were better able to maintain the metronome rate with self-feedback and with other feedback than in the absence of any auditory feedback. Interestingly, musicians were also better able to maintain the metronome rate with self-feedback only and with other feedback only, compared to full feedback. This result could be interpreted as increased noise introduced by two competing auditory signals or, similarly, competition between accepting one’s own auditory feedback as the valid signal and accepting the partner’s auditory feedback as the valid signal when they differed. In contrast, nonmusicians were better able to maintain the beat of the metronome in the presence of self-feedback compared to no feedback and were worse at maintaining the metronome beat in the presence of feedback from their partner, in line with the findings of Konvalinka et al. (2010). This result could be attributed to difficulty keeping the beat in the presence of a temporally variable and unpredictable auditory signal such as that produced by nonmusician partners. In support of this interpretation, nonmusicians also unintentionally synchronized with their partner when their partner’s feedback was available, indicating that participants may have stopped maintaining the metronome rate and, instead, converged with their partner. Therefore, time-keeping abilities of nonmusicians may have been negatively affected by the irregular timing produced by their partner’s feedback. This hypothesis is in line with the results of Repp (2006) that showed that distractors with changing phase relationships can perturb synchronization to an isochronous metronome.

Interpreted through dynamical systems theory (Goldenstein, Large, & Metaxas, 1999), auditory feedback from one’s own actions may result in a self-sustained oscillator; quasi-periodic auditory feedback aids the motor system in maintaining the beat because the feedback approximately resonates at the beat frequency, thus reducing damping (i.e., energy loss at each beat cycle). The oscillator may not be completely self-sustaining but, instead, provides some resistance to disentrainment for some period of time that likely depends on several individual differences (e.g., musical training, motor ability, and working memory for temporal intervals). Future studies could examine the limits of these self-sustaining oscillations and which individual differences facilitate (or hinder) time-keeping abilities. With regard to auditory feedback from a partner, results indicated that externally generated feedback may act as a strong attractor and shift entrainment away from the metronomic beat prescribed in the synchronization phase. Although musicians were able to maintain the beat together in the presence of their partners’ feedback, nonmusicians stopped maintaining the prescribed beat while still synchronizing with their partner. Thus, auditory feedback from a partner was a stronger attractor than the prescribed metronomic rate resulting in instability of continued maintenance of the beat rate when the auditory feedback, or source of auditory feedback, was unreliable.

## Experiment 2

Experiment 2 paired musicians with nonmusicians in the same dyadic tapping paradigm to test the hypothesis that synchronization between nonmusicians was perturbed in Experiment 1 because their nonmusician partners produced auditory feedback with unstable timing. If nonmusicians are able to better maintain the metronome rate in conditions in which they hear a musician partner’s feedback, then this would suggest that nonmusicians have difficulty keeping a metronome rate in the presence of an unreliable temporal signal. Experiment 2 used the same design as Experiment 1, with the single change that each dyad consisted of one musically trained and one musically untrained participant.

### Method

#### Participants

Participants were musicians (*N* = 20) with six or more years of formal training on an instrument (*M* = 11.85 years, SD = 3.83, range = 6 to 17 years) and nonmusicians (*N* = 20) with less than three years of formal training on an instrument (*M* = 0.65 years, SD = 0.75, range = 0 to 2 years) recruited from the McGill University community. Of the musicians, 12 were female (8 male) and the mean age was 23.85 years (SD = 4.04, range 18 to 35 years). Of the nonmusicians, 13 were female (7 male) and the mean age was 24.95 years (SD = 6.82, range 18 to 46 years). No participant reported any hearing impairment. Participants in Experiment 2 were not participants in Experiment 1.

#### Apparatus and procedure

The apparatus and procedure were identical to Experiment 1 except that pairs consisted of one musician and one nonmusician.

#### Statistical analysis

Data were analyzed using the same LMEM as that used for Experiment 1 with the exception that we included musical training (musician, nonmusician) as a between-subjects variable for coordination with the hypothesized metronome but not for coordination with a partner because partners were mixed.

### Results

#### Synchronization with the cued tempo

For the cross-correlations between individuals’ taps and the hypothesized metronome, there were significant main effects of Self-feedback [*F* (1, 415.94) = 16.79, *p* < 0.001, $\eta _{{\text{g}}}^{2}$ = 0.02], Other feedback [*F* (1, 336.82) = 4.87, *p* = 0.02, $\eta _{{\text{g}}}^{2}$ = 0.02], and Musical expertise [*F* (1, 44.55) = 7.16, *p* = 0.01, $\eta _{{\text{g}}}^{2}$ = 0.09]. There was a significant interaction between Self and Other feedback [*F* (1, 419.45) = 13.87, *p* < 0.001, $\eta _{{\text{g}}}^{2}$ = 0.05], and a near-significant interaction between Other feedback and Musical expertise [*F* (1, 155.14) = 3.87, *p* = 0.05, $\eta _{{\text{g}}}^{2}$ = 0.03]. No other interactions reached significance (*ps* > 0.81).

**Fig. 3:**
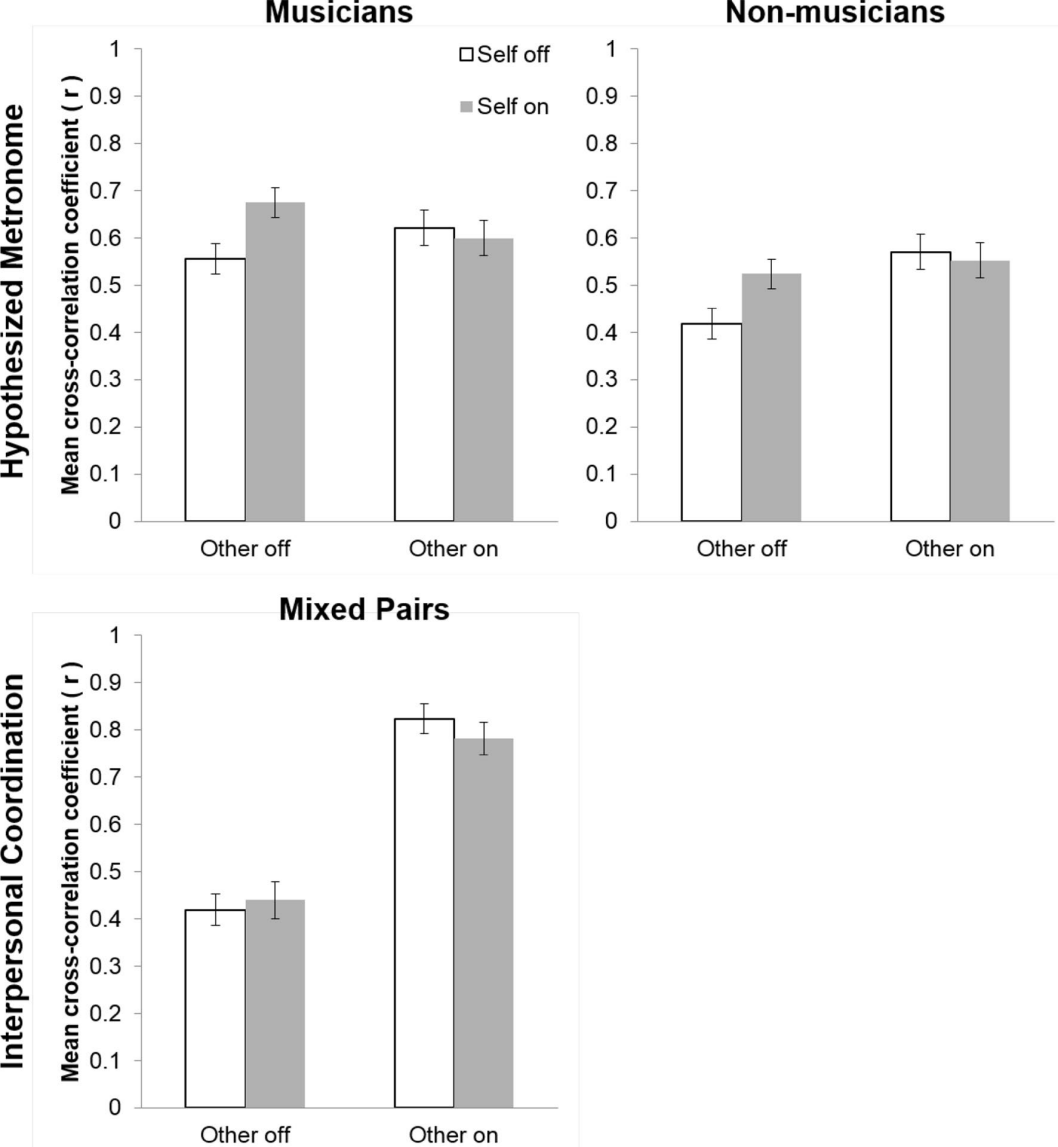
Mean cross-correlation coefficients between wave transformations of the hypothesized metronome and tap onsets for musicians (top left panel) and nonmusicians (top right panel), and between tap onsets of the two participants within each mixed pair (bottom panel) for feedback conditions in Experiment 2

The main effect of musical training indicated that musicians were more coordinated with the hypothesized metronome than nonmusicians (see Fig. 3). As the interaction between Musical expertise and Other feedback approached significance and the effect size was non-trivial, we proceeded with planned comparisons. Musicians had significantly higher cross-correlation coefficients than nonmusicians for the other off condition (*p* = 0.02), but not for the other on condition (*p* = 0.72).

Planned comparisons were conducted to examine the interaction between Self and Other feedback. As shown in Fig. 3 (top panels), the no feedback condition (Self off, Other off) produced cross-correlation coefficients that were significantly smaller than the self-feedback only condition (Self on, Other off; *p* < 0.001) but did not significantly differ from the other feedback only (Self off, Other on; *p* = 0.995) or full feedback (Self on, Other on; *p* = 0.97) conditions. Self-feedback only (Self on, Other off) was significantly smaller than other feedback only (Self off, Other on; *p* < 0.001) and full feedback (Self on, Other on; *p* < 0.001). Other feedback only (Self off, Other on) was not significantly different from full feedback (Self on, Other on; *p* = 0.99). These results support the hypothesis that musicians and nonmusicians use auditory feedback from themselves and from a partner to maintain a regular beat.

#### Interpersonal coordination

For the cross-correlations between partners’ taps, there was a near-significant main effect of Self-feedback [*F* (1, 448.55) = 3.22, *p* = 0.07, $\eta _{{\text{g}}}^{2}$ = 0.002], a significant main effect of Other feedback [*F* (1, 447.18) = 940.78.79, *p* < 0.001, $\eta _{{\text{g}}}^{2}$ = 0.61], and a significant interaction between Self and Other feedback [*F* (1, 454.35) = 6.41, *p* = 0.01, $\eta _{{\text{g}}}^{2}$ = 0.01]. As shown in Fig. 3 (bottom panel), the significant interaction between Self and Other feedback indicates that cross-correlation coefficients were significantly greater in both Other feedback on conditions (full feedback and other feedback only) than in Other feedback off conditions (self-feedback only and no feedback conditions; *ps* < 0.001). Planned comparisons further revealed that cross-correlations coefficients were significantly greater for the other only feedback condition (Self off, Other on) compared to the full feedback condition (Self on, Other on; *p* = 0.01); no feedback (Self off, Other on) and self-feedback only (Self on, Other off) did not significantly differ (*p* = 0.95). These results support the hypothesis that musicians and nonmusicians unintentionally synchronize with the sound productions of a partner, even when not instructed to do so.

### Discussion

Results of Experiment 2 showed that when musicians are paired with nonmusicians, both partners are better able to maintain the rate of the metronome with self-feedback only, other feedback only, and full feedback relative to no feedback. Moreover, partners unintentionally synchronized with each other when they could hear their partner’s sound productions. These results are in line with those of the musician pairs in Experiment 1. Taken together, the results of Experiments 1 and 2 indicate that nonmusicians can use the feedback provided by musicians to better maintain the metronomic rate or tempo. Konvalinka et al. (2010) found that participants were significantly worse at keeping a cued tempo in the presence of auditory feedback from a partner. The present results suggest that auditory feedback from a partner only perturbed entrainment when both partners lack musical expertise. However, a comparison of the pairs in Experiment 1 (nonmusician pairs and musician pairs) and Experiment 2 (mixed pairs) is in order.

### Comparison of Experiments 1 and 2

To statistically test the interpretation that nonmusicians were better able to maintain the metronome rate with musicians than with other nonmusicians, we pooled the metronome synchronization data from the two experiments in an LMEM with fixed factors Self-feedback (2; off, on) and Other feedback (2; off, on) with Musical expertise and Pair type (same, Experiment 1 and mixed, Experiment 2) as between-subjects factors, and random effects participant nested within pair and tempo (3; fast, medium, slow). As in Experiments 1 and 2, the cross-correlation coefficient representing synchronization with their partner was entered as a predictor variable within pairs. There were significant interactions between Other feedback, Musical expertise, and Pair type [*F* (1, 482.76) = 23.96, *p* < 0.001, $\eta _{{\text{g}}}^{2}$ = 0.04], Self-feedback, Other feedback, and Musical expertise [*F* (1, 1256.62) = 7.07, *p* = 0.007, $\eta _{{\text{g}}}^{2}$ = 0.007], and a significant four-way interaction between Self-feedback, Other feedback, Pair type, and Musical expertise [*F* (1, 1256.62) = 5.26, *p* = 0.02, $\eta _{{\text{g}}}^{2}$ = 0.005]. The interaction between Musical expertise and Pair type approached significance [*F* (1, 116.77) = 3.65, *p* = 0.06, $\eta _{{\text{g}}}^{2}$ = 0.005]; no other interactions with Pair type or Musical expertise were significant (*p*s > 0.13).

Planned comparisons were performed to examine whether nonmusicians were significantly better at maintaining the tempo when paired with musicians than with nonmusicians, and to test whether musicians were significantly worse at maintaining the tempo when paired with nonmusicians than with musicians. In both Other feedback on conditions (other feedback only, full feedback), nonmusicians paired with musicians were significantly better at maintaining the tempo than nonmusicians paired with nonmusicians (*p*s < 0.007). Nonmusicians paired with musicians did not significantly differ from nonmusicians paired with nonmusicians in the no feedback condition (Self off, Other off; *p* = 0.19) or the self-feedback only condition (Self on, Other off; *p* = 0.99). Musicians paired with nonmusicians were not significantly worse than musicians paired with musicians for any feedback condition (*p*s > 0.17). Thus, nonmusicians were better able to keep the beat in the presence of a musician’s sound productions and musicians were not significantly hindered by the presence of a nonmusician’s sound productions.

## General discussion

Two experiments investigated the role of auditory feedback and musical expertise in dyadic tapping. The principal findings were that both musicians and nonmusicians used auditory feedback from their own sound productions to maintain the beat. In addition, both groups unintentionally synchronized with their partner even though they were not instructed to do so, regardless of whether their partner was accurately maintaining the tempo. Nonmusicians in nonmusician pairs were significantly worse at keeping the beat when they could hear their fellow nonmusician’s sound productions. Musicians in musician pairs were able to use their partner’s sound productions to maintain the beat but were slightly worse when they could simultaneously hear their own sound productions. Interestingly, this difference between other feedback only and full feedback was not evident for nonmusician pairs or for musicians paired with nonmusicians, indicating that paired musicians may have been competing for leader–follower roles (Goebl & Palmer, 2009). It is possible that if leader and follower roles were assigned to the two participants, then there would be no difference between the other feedback only and full feedback conditions for musicians. As the present study focused on keeping the beat (and not explicit synchronization) imposing leader and follower roles would have defeated its purpose. Future experiments could impose leader–follower roles to examine how leaders and followers may differentially use the sound productions of self and other to maintain the tempo.

A novel finding was that nonmusicians were better able to maintain a consistent tempo when paired with expert musicians, compared to nonmusicians. If such timing improvements transfer to music performance, then it seems likely that novice music students can pedagogically benefit from performing simultaneously with expert musicians. Whether the timing improvement in students would remain in the absence of the expert musicians, however, remains to be empirically tested. It may still be the case that students or novice performers (e.g., dancers or musicians) are able to benefit or temporarily improve their performance timing by playing in music ensembles that consist of one or more expert musicians. Duet performances between novices and expert performers (pianists) has, to our knowledge, only been examined in one study (Wolf, Sebanz, & Knoblich, 2018). Wolf et al. found that asynchronies between keystroke onsets of experts and novices were smaller in absolute magnitude and less variable when experts were familiar with the novice’s performance for easy passages but not for difficult passages (where familiarity with the score contributed to smaller and less variable asynchronies). Longitudinal studies that track practice effects with novices and experts have not yet been reported and could elucidate whether expert performers can facilitate the performance of novices with short-term and long-term benefits for timing abilities.

Unintentional synchronization between partners was evident for musician pairs, nonmusician pairs, and mixed pairs. These results are in line with those of Repp (2006) who found that synchronization with external signals can be influenced by irregular distractors that compel a person to coordinate their actions with the timing of the distractor signal. Moreover, the process by which dyads coordinate their actions appears to be automatic and, as shown in the present study, occurs even under the instruction to maintain the given tempo. Previous research has indicated that interpersonal coordination and synchrony increases affiliation (Hove & Risen, 2009); it may be that this automatic process stems from the evolutionary advantage offered by strong group bonds. In other words, although participants were instructed to maintain the given tempo, a biological instinct to adapt may have encouraged them to synchronize with one another. Experiments using virtual partners (e.g., Van Der Steen & Keller, 2013) that are known to be non-humans or are presented under the guise of real participants in dyadic tapping could disentangle the social factors that lead to spontaneous synchronization. If social cohesion drives spontaneous synchronization (intentional or otherwise), then humans would synchronize less with a known non-human than with a partner they believe to be human. Launay, Dean, and Bailes (2014) have shown that people demonstrate greater likeability of virtual partners that are more synchronous when they believe the partner is human, but likeability did not change with synchrony when participants knew they were performing with a computer.

Synchrony with a virtual partner has also been examined in a study that manipulated knowledge of their partner’s humanness (Mills, Harry, Stevens, Knoblich, & Keller, 2018). Results indicated that synchrony with a virtual partner thought to be human was not significantly better than that with a virtual partner thought to be a computer. However, the degree of adaptivity of the virtual partner and subjective rating of “easiness” of the humanness conditions (“human” or “computer” virtual partner) interacted with the manipulation of humanness. Those who found the “human” virtual partner condition easier synchronized more in the moderately adaptive condition (compared to low adaptivity) for only the “human” virtual partner. Those who found the “computer” virtual partner condition synchronized more in the moderately adaptive condition (compared to low adaptivity) for only the “computer” virtual partner. The group with no preference synchronized more in the moderately adaptive condition (compared to low adaptivity) for both virtual partners. Overall, these results indicate that perceived humanness on a partner can influence coordination, but it has a complicated relationship with perceived difficulty and other individual differences that requires further exploration.

Interpreted though the dynamic attending theory (Jones & Boltz, 1989) and dynamical systems theory (Goldenstein et al., 1999), results of the present study indicate that a somewhat reliable external auditory signal, such as feedback from one’s self or feedback from a partner, can aid in maintaining a beat through entrainment. The present results suggest that, in the absence of auditory feedback, neural oscillations entrained to the cued metronome are not self-sustaining and quickly lose the beat, perhaps returning to a natural or resting frequency (see Loehr & Palmer, 2011). Conversely, auditory feedback from one’s own actions can act to regulate timing and provides an auditory signal that may aid regular neural oscillations at the cued tempo, at least for a period of time. Auditory feedback from a partner can also aid in sustaining neural oscillations but only if the partner is producing a regular signal with the correct period. In the case of nonmusician pairs, nonmusicians abandoned the prescribed tempo and, instead, synchronized with their partner. In other words, in the absence of a stable periodic beat, nonmusicians may have been unable to sustain the neural oscillations that were entrained to the metronomic rate and, instead, became entrained to the auditory feedback of their partner. This interpretation is corroborated by the results of dyadic tapping experiments in which there is little coordination between performers when the metronome persists throughout the trial (Mates et al., 1992; Nowicki et al., 2013). These studies show strong synchrony with the metronome and weak mutual adaptation between partners when auditory feedback of the partner is presented alongside the stable metronome. The interpretation of unstable neural oscillations in the presence of an unreliable auditory signal is further supported by the results of Repp (2006) that show even musicians can “lose the beat” if they are sufficiently perturbed by an auditory distractor sequence. The present study suggests that under conditions of bidirectional feedback (i.e., feedback from both partners) it is possible for the neural oscillations of partners to become coupled and oscillator prevent oscillator damping for the cued tempo if the sound productions of at least one partner are relatively stable. Furthermore, musicians can function as a strong attractor; auditory feedback from a musician partner improved the entrainment of nonmusicians without any significant detriment to the entrainment of the musician.

## Conclusions

We demonstrated that musicians and nonmusicians use auditory feedback from their own sound productions and the sound productions of a partner to maintain a tempo. Nonmusicians were better at keeping the beat in the presence of auditory feedback from an expert musician compared to feedback from a partner who lacked musical expertise. Moreover, musicians and nonmusicians unintentionally synchronized when they could hear their partner regardless of whether their partner was a musician or a nonmusician. Our findings indicate that nonmusicians (or novice musicians) undergoing musical training may benefit from the strong time-keeping abilities of expert musicians in musical ensembles and might be hindered when performing with other nonmusicians or novices. Long-term pedagogical benefits for novice music students who perform with expert musicians provide an exciting new avenue for future research. These findings may further generalize to any skill that requires synchronization and/or fine timing, such as, team sports (e.g., rowing).

## Footnotes

1 A less conservative analysis that did not use the synchronization with a partner as a predictor variable in the LMEM demonstrated significant differences between all feedback conditions (*p*s < 0.002) except between self-feedback only (Self on, Other off) and other feedback only (Self off, Other on; *p* = 0.68), and between self-feedback only (Self on, Other off) and full feedback (Self on, Other on, *p* = 0.06). Given that partner synchronization should only influence an individual’s synchronization with the metronome in conditions where other feedback is on, it is possible that the conservative analysis reported here masks significant differences between Other feedback conditions (Other on and off) for Musician pairs in Experiment 1. Other effects reported in Experiments 1 and 2 produced similar patterns of results for the LMEMs that included and excluded partner synchronization as a predictor variable.
